# The Syk inhibitor R406 is a modulator of P-glycoprotein (*ABCB1*)-mediated multidrug resistance

**DOI:** 10.1371/journal.pone.0210879

**Published:** 2019-01-22

**Authors:** George E. Duran, Branimir I. Sikic

**Affiliations:** Department of Medicine, Division of Oncology, Stanford University School of Medicine, Stanford, California, United States of America; Wayne State University, UNITED STATES

## Abstract

In a previously published study, higher levels of spleen tyrosine kinase (Syk) were observed in recurrent post-chemotherapy ovarian cancers compared to primary tumors. Syk inhibition was found to stabilize microtubules and potentiate paclitaxel activity in cellular models of taxane-resistant ovarian cancers. We further studied the effects of Syk inhibition on paclitaxel activity in Syk(+) ovarian cancer cell models and in variants selected for taxane resistance. Syk inhibition was accomplished using RNAi and by exposure to the small molecule competitive inhibitor R406, the active metabolite of fostamatinib. Exposure to R406 or to a *SYK*-specific pool of siRNAs did not alter taxane activity in the OVCAR-3 cell line, which has the most Syk content in our panel of nine human ovarian cancer cell lines. However, treatment with R406 sensitised the multidrug resistant (MDR) variants MES-SA/Dx5 and SK-OV-3/TR to paclitaxel in a dose-dependent manner resulting from the inhibition of the *ABCB1*/P-glycoprotein (P-gp) drug transporter. These observations are Syk-independent since both MDR cell models are Syk negative. R406 modulated resistance to other known P-gp substrates, and we observed orthovanadate-sensitive ATPase stimulation resulting from treatment with R406. These data indicate that the chemo-sensitizing effect of R406 in taxane-resistant cells previously reported was not associated with Syk but resulted from the modulation of P-gp-mediated MDR.

## Introduction

The taxanes, paclitaxel (Taxol), docetaxel (Taxotere), and the second-generation taxane cabazitaxel (Jevtana), are used to treat breast, ovarian, lung, prostate and other cancers. These potent anticancer agents stabilize microtubules, blocking cells in the late G2/M phase of the cell cycle [1]. Pre-existing or acquired drug resistance during the course of therapy affects their clinical efficacy. A major determinant of taxane activity is the multidrug resistance transporter P-gp, and *in vitro* drug selections with taxanes alone result in the activation of the *ABCB1* gene [2]. This resistance can be modulated in the presence of known transport inhibitors such as the PSC-833 (valspodar) and LY335979 (zosuquidar), among others [3, 4].

Among its many targets, spleen tyrosine kinase (Syk) can bind microtubules and other proteins associated with the cytoskeleton [5–7]. Recent published studies have implicated a role for Syk in determining the response to taxane treatment in human ovarian cancer cell models [8], with one study demonstrating improved paclitaxel activity in taxane-selected variants treated with the Syk inhibitor R406 [9]. We studied the effects of inhibiting Syk on taxane activity in OVCAR-3, which expresses the most total Syk and phospho-Syk in our panel of nine human ovarian carcinoma cell lines. Silencing *SYK* using a pool of four specific siRNAs and treatment with R406 did not affect taxane sensitivity, nor did we observe any effects on the cell cycle by FACS or on tubulin polymerization in response to taxane treatment compared to non-targeting controls. We were able to reproduce the modulation of taxane resistance in a model of MDR and confirmed that R406 is an inhibitor of the P-gp transporter.

## Methods

### Drugs and reagents

The anticancer drugs doxorubicin, paclitaxel, and vinblastine were obtained from the drug repository of the National Cancer Institute (Bethesda, MD). Docetaxel was a gift from Sanofi Oncology (Bridgewater, NJ). Novartis Pharmaceuticals (East Hanover, NJ) kindly provided the P-gp inhibitor PSC-833 (valspodar), and LY335979 (zosuquidar) was a gift from Kanisa Pharmaceuticals (San Diego, CA). All drugs were prepared in 100% ethanol as 1 mmol/L stock solutions and stored at -20 °C. The anti-Syk inhibitor R406 was purchased from Selleck Chemicals (Houston, TX) as a 10 mmol/L stock in DMSO.

### Cell culture

The OVCAR-3 and SK-OV-3 human ovarian adenocarcinoma cell lines were purchased from the American Type Culture Collection (ATCC, Manassas, VA). The human ovarian clear cell carcinoma cell line ES-2 and human ovarian carcinoma MES-OV were established in our laboratory (all authenticated and available at the ATCC as CRL-1978 and CRL-3272, respectively). OVCA429 and OVCA433 cells were a gift from Dr. Robert Bast (The University of Texas MD Anderson Cancer Center, Houston, TX), and OVCAR-4, OVCAR-5, and IGROV-1 were provided by the National Cancer Institute’s tumor repository (Bethesda, MD).

A second parental SK-OV-3 cell line and its paclitaxel-selected SK-OV-3/TR variant were obtained from Dr. le-Ming Shih (Johns Hopkins University School of Medicine, Baltimore, MD). Syk expression and taxane sensitivity was comparable to the parental SK-OV-3 purchased from the ATCC. The human uterine sarcoma cell line MES-SA was established in our laboratory (ATCC, CRL-1976). Its doxorubicin-selected human uterine sarcoma *ABCB1* variant MES-SA/Dx5 (ATCC, CRL-1977) was used as a positive control for transporter activity and demonstrates a typical MDR phenotype [10].

Cells were grown in McCoy 5A medium supplemented with 10% (v/v) fetal bovine serum, 100 U of penicillin/mL, and 100 μg of streptomycin/mL (Corning Life Sciences, Tewksbury, MA) at 37 °C in a humidified atmosphere containing 5% CO_2_, and were routinely screened to rule out mycoplasma infection.

### Growth inhibition assays

The *in vitro* activity of various anticancer drugs was tested using a modified sulforhodamine B (SRB) colorimetric assay following a 72 h drug incubation representing approximately three cell divisions [11]. Drug effects were calculated as a percentage relative to untreated control survival, and response versus drug concentration was calculated using the Hill equation in KaleidaGraph software (Synergy Software, Reading, PA). Each drug concentration was tested in quadruplicate measurements per experiment.

### Western blotting

Total protein lysates were isolated from growing cells using 1x radioimmunoprecipitation assay buffer [RIPA, 1% (v/v) NP40, 0.5% (w/v) sodium deoxycholate, 0.1% (w/v) SDS in 1x PBS buffer] with freshly added protease inhibitors (cocktail from Bio-Rad Laboratories, Hercules, CA). Total protein (10–30 μg) was separated by 4–20% (w/v) gradient polyacrylamide gels and transferred onto nitrocellulose membranes using the Trans-Blot Turbo transfer system (all Bio-Rad Laboratories). Membranes were blocked overnight at 4 °C in 1x TBS containing 5% (w/v) nonfat milk and 1% (w/v) bovine serum albumin, and then incubated with the following antibodies: anti-P-gp (clone C219, EMD Millipore, Billerica, MA), and specific antibodies for total Syk (D3Z1E), p-Syk (Tyr525/526), and GAPDH (all Cell Signaling Technology, Danvers, MA). These primary antibodies were recognized by species-appropriate horseradish peroxidase-conjugated secondary antibodies, and detected using the Clarity Western ECL substrate on a ChemiDoc System (Bio-Rad).

### Cell cycle analysis following Syk inhibition

A propidium iodide-based assay was used to determine cell cycle changes following taxane treatment with and without exposure to R406. The BD Cycletest Plus DNA Reagent Kit was used according to the manufacturer’s protocol and samples run on a BD LSR II flow cytometer at Stanford’s Shared FACS Facility (BD Biosciences, San Jose, CA).

### Functional assays for transporter activity

Cellular drug accumulation was determined using a published method [12]. Briefly, 1 x 10^6^ cells were seeded in six-well dishes and allowed to attach overnight. [^3^H]-docetaxel (10 nM, American Radiolabeled Chemicals, St. Louis, MO) was allowed to accumulate for 1 h at 37 °C with and without PSC-833 (2 μmol/L) or R406 (1–10 μmol/L), aspirated, and dishes were washed once with ice-cold PBS. Cells were lysed immediately using a 2% (w/v) SDS solution, and counts were determined upon the addition of EcoLite liquid scintillation cocktail (MP Biomedicals, Solon, OH), and normalized to protein content.

These data were confirmed by determining the accumulation of rhodamine-123 and BODIPY-paclitaxel (both Thermo Fisher Scientific, Waltham, MA) by flow cytometry. Cells were harvested, exposed to either rhodamine-123 or BODIPY-paclitaxel for 1 h at 37 °C, drug was removed by centrifugation (200 x *g*) at 4 °C, and cells washed once in cold PBS. In order to correlate drug accumulation with P-gp content, cells were stained on ice using an anti-P-gp mouse monoclonal which detects an external epitope (clone UIC2, EMD Millipore, Billerica, MA), and detected by a Texas Red goat anti-mouse secondary antibody (Thermo Fisher Scientific) using an LSR II flow cytometer. The effects of PSC-833 (2 μmol/L) and R406 (0.5–10 μmol/L) were accessed in separate experimental conditions.

### Transient *SYK* silencing by small interfering RNA

Pools of four *SYK*-specific siRNAs were designed using Dharmacon’s siDesign Center algorithm and synthesized (ON-TARGET*plus* reagents, GE Dharmacon, Lafayette, CO). Lipid-mediated siRNA delivery into cells was accomplished with DharmaFECT 1 transfection reagent (GE Dharmacon) according to the manufacturer’s protocol 24 h after cells were seeded. Cells were allowed to incubate in siRNAs for 24 h prior to drug treatment. Optimal concentrations of siRNAs were determined in order to avoid off-target effects, and the time course of gene silencing was evaluated relative to non-targeting controls (GE Dharmacon) from 24 to 96 h after transfection by RT-qPCR and immunoblotting with specific antibodies.

### Drug-stimulated ATPase activity

ATPase stimulation was measured using the Corning Gentest ATPase assay (BD Biosciences, Woburn, MA) in membranes isolated from insect cells (BTI-TN5B1-4) infected with human *ABCB1* cDNA using a baculovirus expression system (BD Biosciences). Membranes were incubated with drug for 5 min at 37 °C in the presence and absence of ATP, followed by an additional 30 min incubation prior to the addition of a colorimetric reagent according to the manufacturer’s protocol. All experimental conditions were run with and without sodium orthovanadate, and microtiter plates were read at 800 nm on a Spectramax Paradigm (Molecular Devices, Sunnyvale, CA).

## Results

### Syk inhibition by RNAi or treatment with R406 does not affect taxane activity

**Fig 1:**
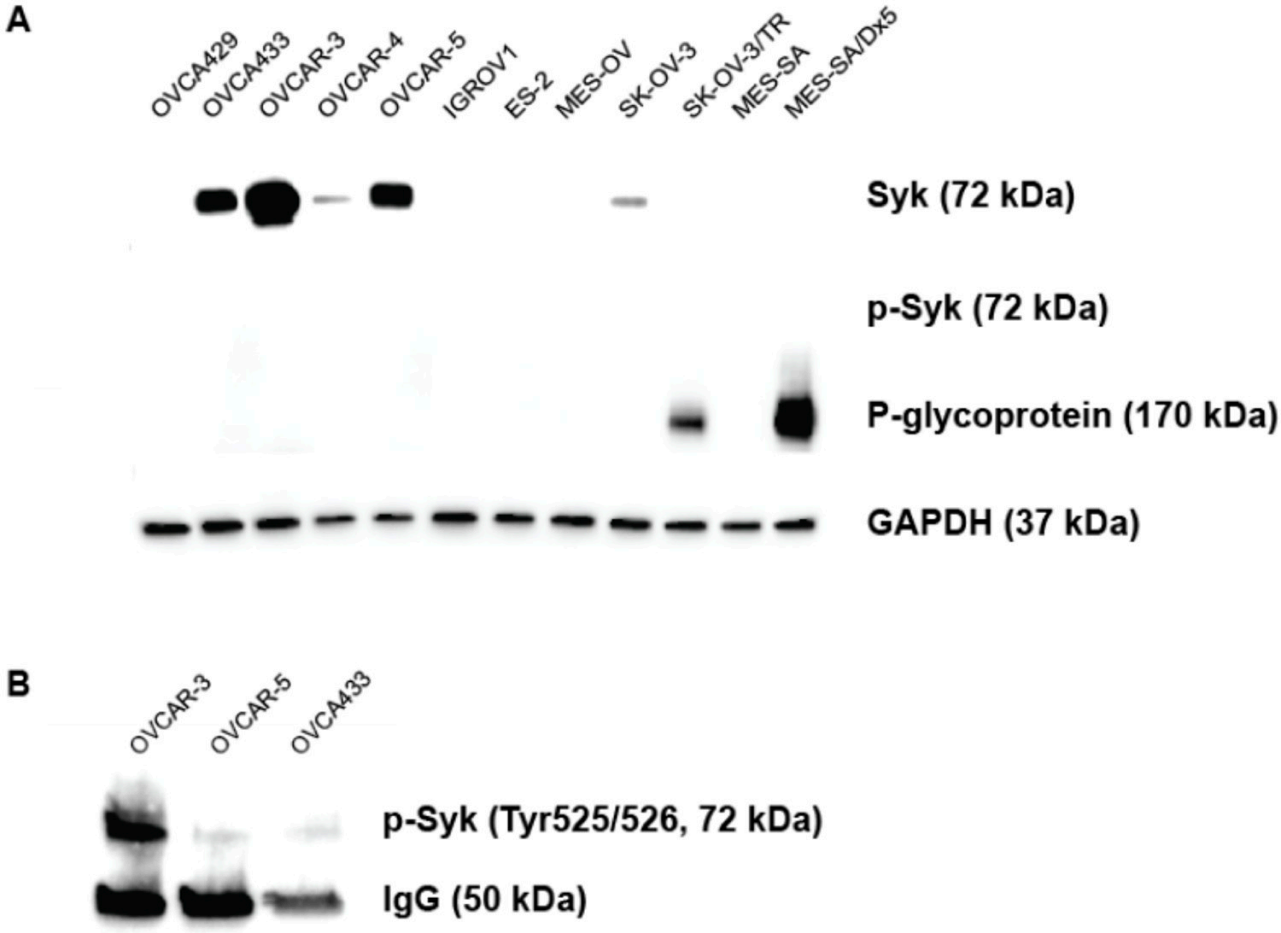
OVCAR-3 expresses the most Syk in a panel of nine human ovarian cancer cell lines. (A) Syk expression was determined by immunoblotting using a specific rabbit polyclonal antibody. Images captured on a ChemiDoc System (Bio-Rad) are shown and a longer exposure (>600 s) revealed a faint Syk band in IGROV-1 cells. Parental SK-OV-3 cells and its MDR variant SK-OV-3/TR, along with the human uterine sarcoma MES-SA and its doxorubicin-selected MES-SA/Dx5 variant were included, and P-gp status determined using the C219 monoclonal antibody. No p-Syk was detected in total cell lysates. 30 μg of protein was loaded onto each lane and an anti-GAPDH antibody was used to confirm uniform loading. (B) Total Syk was immunoprecipated and then screened for p-Syk expression in the three most Syk(+) cell lines: OVCAR-3, OVCAR-5, and OVCA433.

Syk expression was determined by immunoblotting in a panel of nine human ovarian cancer cell models. Five cell lines (OVCA433, OVCAR-3, OVCAR-4, OVCAR-5, and SK-OV-3) were positive (Fig 1A), while a faint band was observed in IGROV-1 with a longer exposure time, and the remaining cell lines were negative under identical experimental conditions. Staining cells with the same rabbit monoclonal confirmed the Syk expression levels by flow cytometry. Phospho-Syk (p-Syk, Tyr525/526) levels were determined by immunoprecipitation in the cell lines with the most total Syk content (OVCA433, OVCAR-3, and OVCAR-5), and a strong band was present in OVCAR-3 cells (Fig 1B).

**Fig 2:**
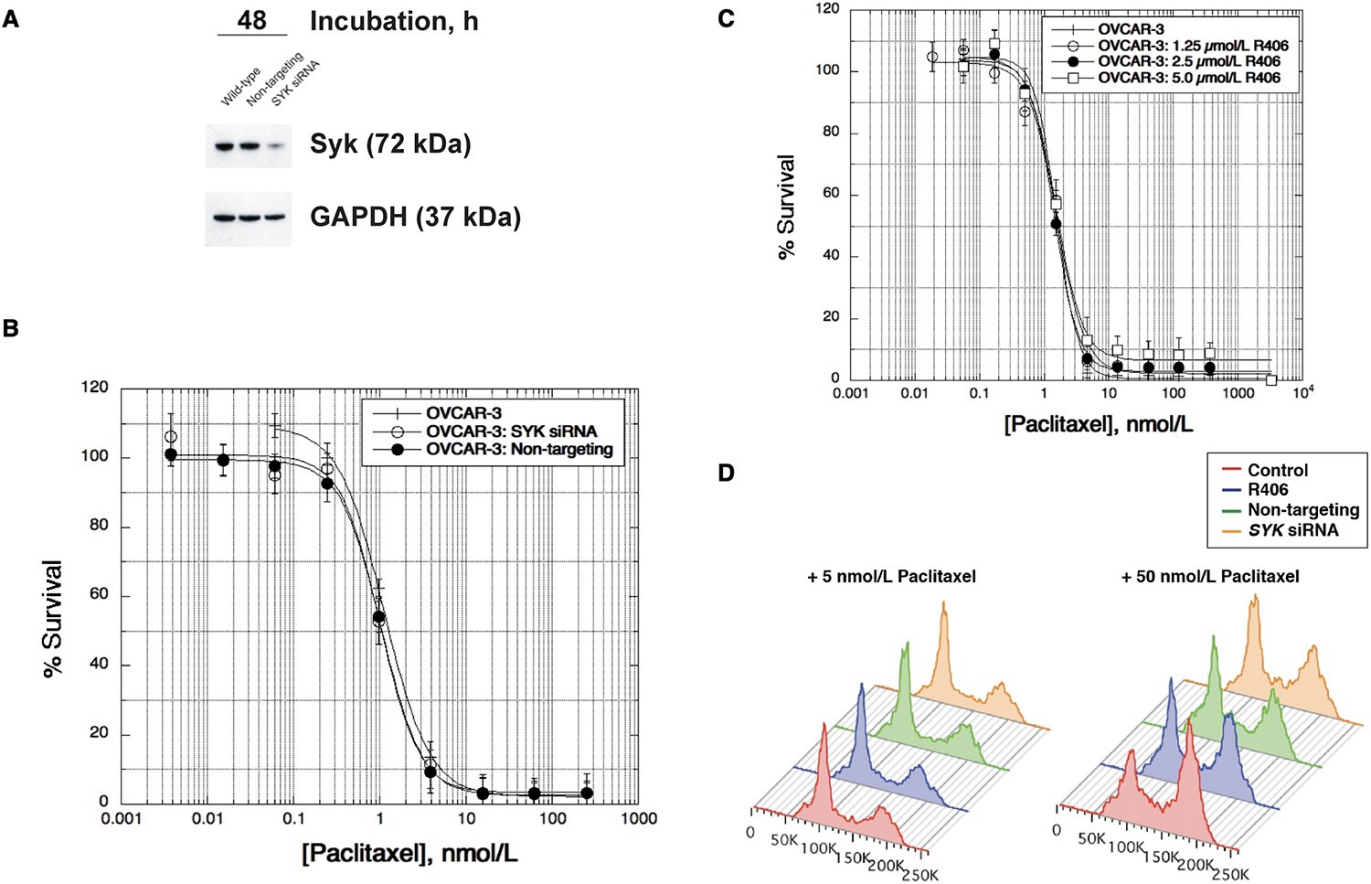
Syk inhibition does not affect taxane activity in OVCAR-3. (A) OVCAR-3 cells were transfected with a *SYK*-specific SMARTpool and screened for Syk expression by immunoblotting compared to wild-type and cells transfected with a non-targeting control SMARTpool. (B) Paclitaxel was added 24 h post-transfection and activity screened by SRB assay following a 72 h drug incubation representing approximately three cell divisions. (C) Paclitaxel sensitivity was determined in OVCAR-3 cells following exposure to R406 (1.25–5 μmol/L) and read at 72 h. (D) Following a 24 h paclitaxel incubation (5 or 50 nmol/L), OVCAR-3 cells transfected with either the non-targeting control, *SYK*-specific siRNA pool, or exposed to R406 (5 μmol/L) were screened for cell cycle analysis by flow cytometry.

In order to evaluate the functional significance of Syk expression, we employed two methods: silencing *SYK* using RNAi and inhibiting Syk using the potent small molecule R406. A Dharmacon SMARTpool of four *SYK*-specific siRNAs was transfected into OVCAR-3, and greater than 90% silencing was achieved over a period of 72 h compared to untreated cells and cells transfected with a pool of non-targeting siRNAs. Fig 2A presents data 48 h post-transfection in cells tested for taxane activity. No significant difference in either paclitaxel (Fig 2B) or docetaxel activity was observed when comparing the IC_50_ values for the untreated OVCAR-3 cells to cells transfected with either the non-targeting control or the *SYK*-specific SMARTpools. We confirmed this result by exposing OVCAR-3 cells to various concentrations of the Syk inhibitor R406 (1.25 to 5 μmol/L) and screened for paclitaxel activity. There was no significant difference in IC_50_ values comparing cells exposed to R406 and the untreated control (Fig 2C).

We further explored possible Syk effects by analyzing cell cycle staining patterns following a dose of paclitaxel (24 h, 5 and 50 nmol/L) in cells treated with R406 or transfected with the anti-*SYK* siRNA pool, and we did not detect any differences compared to control OVCAR-3 cells using flow cytometry (Fig 2D). Separate FACS assays screened cells for Annexin V-FITC expression, and there was no difference in the percentage of apoptotic cells detected in the R406 treated cells compared to cells treated with paclitaxel alone.

### R406 modulates P-gp-mediated drug resistance

Yu et al. observed significant taxane sensitization upon treatment with R406 in the paclitaxel-selected variant SK-OV-3/TR, which is known to express the P-gp transporter [13–15]. We confirmed P-gp expression and activity in this MDR variant, but unlike the authors we found that the SK-OV-3/TR cell line was negative for Syk by immunoblotting (Fig 1A). We confirmed the ability of R406 to modulate paclitaxel resistance in SK-OV-3/TR with complete sensitization achieved at 2.5 μmol/L R406 in SRB colorimetric sensitivity assays (Table 1). The known P-gp inhibitors PSC-833 (2 μmol/L) and LY335979 (300 nmol/L) were included in these assays as positive controls for MDR modulation, and we achieved complete sensitization of paclitaxel resistance in SK-OV-3/TR cells (Table 1). Treatment with R406 in the Syk(+) SK-OV-3 parental cell line had no effect on paclitaxel activity.

**Table 1:** The effect of the Syk inhibitor R406 on paclitaxel resistance in SK-OV-3/TR. SRB colorimetric assays were run following 72 h drug incubations and data expressed as the mean of quadruplicate determinations. The P-gp inhibitors PSC-833 (PSC) and LY335979 (LY) were included as positive controls for MDR modulation.

		IC_50_^1^ (Modulation Ratio^2^)/Relative Resistance^3^
		Paclitaxel
**SK-OV-3**		
	Control	2.1 ± 0.23
	+ 0.25 μmol/L R406	2.0 ± 0.15 (1.0)
	+ 0.5 μmol/L R406	1.8 ± 0.34 (1.2)
	+ 1 μmol/L R406	1.9 ± 0.28 (1.1)
	+ 2.5 μmol/L R406	2.0 ± 0.12 (1.0)
	+ 2 μmol/L PSC	1.7 ± 0.33 (1.2)
	+ 300 nmol/L LY	1.8 ± 0.26 (1.2)
**SK-OV-3/TR**		
	Control	35 ± 4.1 /17
	+ 0.25 μmol/L R406	15 ± 1.8 (2.3)/7.5
	+ 0.5 μmol/L R406	3.8 ± 0.46 (9.2)/2.1
	+ 1 μmol/L R406	3.2 ± 0.44 (11)/1.7
	+ 2.5 μmol/L R406	2.2 ± 0.27 (16)/1.1
	+ 2 μmol/L PSC	2.3 ± 0.39 (15)/1.3
	+ 300 nmol/L LY	2.0 ± 0.23 (17)/1.1

^1^ The IC_50_ value (nmol/L) is the drug concentration required to kill 50% of the population and is calculated from semi-logarithmic growth curves, with data expressed as mean values ± standard deviations.

^2^ Modulation ratios are calculated by dividing the IC_50_ value of MDR variant by the IC_50_ of the variant treated with various concentrations of small molecule inhibitor.

^3^ The relative resistance was calculated by dividing the IC_50_ value of MDR variant alone by the IC_50_ of the parental cell line under the same experimental conditions.

**Fig 3:**
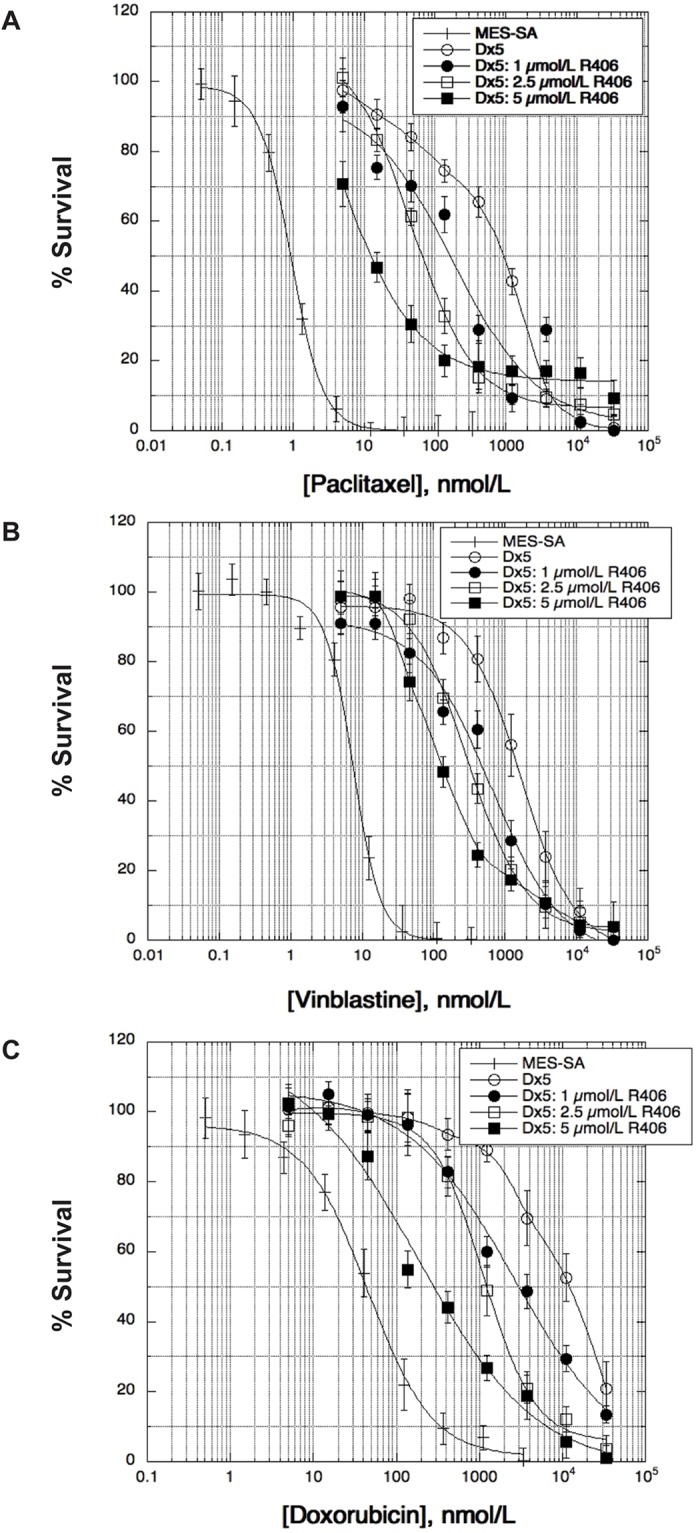
R406 modulates resistance to P-gp substrates. The effect of R406 (1–5 μmol/L) on three P-gp substrates, paclitaxel (A), vinblastine (B), and doxorubicin (C) in the MDR variant MES-SA/Dx5 was determined by SRB colorimetric assays following 72 h drug incubations.

We tested the ability of R406 to modulate taxane resistance in another MDR model, the doxorubicin-selected human uterine sarcoma variant MES-SA/Dx5, which expresses higher levels of transporter than SK-OV-3/TR (8-fold) and is negative for Syk expression (Fig 1A). Although we did not observe any sensitization in the *ABCB1*(-) parental MES-SA cell line, there was a 5-fold modulation of paclitaxel resistance at 1 μmol/L R406, 14-fold at 2.5 μmol/L, and 69-fold at 5 μmol/L in MES-SA/Dx5 cells that were approximately 900-fold resistant to the drug (Fig 3A, Table 2). Complete modulation of paclitaxel resistance was not possible in this MDR variant since we were not able to achieve concentrations above 5 μmol/L R406 in SRB assays due to the toxic effects of the diluent DMSO.

**Table 2:** The effect of the Syk inhibitor R406 on the activity of three P-gp substrates in MES-SA/Dx5. SRB colorimetric assays were run following 72 h drug incubations and data expressed as the mean of quadruplicate determinations. PSC-833 (PSC) and LY335979 (LY) were included as positive controls for MDR modulation.

		IC_50_^1^ (Modulation Ratio^2^)/Relative Resistance^3^		
		Paclitaxel	Vinblastine	Doxorubicin
**MES-SA**				
	Control	0.95 ± 0.14	7.5 ± 0.93	48 ± 5.5
	+ 1 μmol/L R406	1.5 ± 0.28 (0.63)	7.5 ± 1.1 (1.0)	30 ± 3.5 (1.6)
	+ 2.5 μmol/L R406	0.60 ± 0.11 (1.6)	6.5 ± 1.3 (1.1)	30 ± 5.8 (1.6)
	+ 5 μmol/L R406	0.60 ± 0.90 (1.6)	5.5 ± 0.89 (1.4)	38 ± 6.8 (1.3)
	+ 2 μmol/L PSC	0.80 ± 0.13 (1.2)	6.5 ± 0.87 (1.1)	30 ± 4.4 (1.6)
	+ 300 nmol/L LY	0.91 ± 0.17 (1.0)	6.5 ± 1.2 (1.1)	35 ± 4.5 (1.4)
**MES-SA/Dx5**				
	Control	900 ± 150 /950	1,600 ± 220/210	10,200 ± 890/210
	+ 1 μmol/L R406	180 ± 30 (5.0)/120	650 ± 95 (2.5)/87	2,800 ± 260 (3.6)/58
	+ 2.5 μmol/L R406	65 ± 11 (14)/110	320 ± 45 (5.0)/49	1,200 ± 95 (8.5)/40
	+ 5 μmol/L R406	13 ± 2.1 (69)/22	130 ± 11 (12)/24	280 ± 35 (36)/5.8
	+ 2 μmol/L PSC	1.1 ± 0.18 (820)/1.4	9.8 ± 1.3 (160)/1.5	55 ± 9.5 (190)/1.8
	+ 300 nmol/L LY	0.98 ± 0.16 (920)/1.1	8.1 ± 0.98 (200)/1.2	50 ± 8.5 (200)/1.4

^1^ The IC_50_ value (nmol/L) is the drug concentration required to kill 50% of the population and is calculated from semi-logarithmic growth curves, with data expressed as mean values ± standard deviations.

^2^ Modulation ratios are calculated by dividing the IC_50_ value of MDR variant by the IC_50_ of the variant treated with various concentrations of small molecule inhibitor.

^3^ The relative resistance was calculated by dividing the IC_50_ value of MDR variant alone by the IC_50_ of the parental cell line under the same experimental conditions.

Two additional P-gp substrates were tested in MES-SA/Dx5 including the tubulin depolymerizing agent vinblastine, and the DNA intercalator and Topoisomerase II inhibitor doxorubicin. Treatment with R406 partially sensitized vinblastine and doxorubicin resistance in MES-SA/Dx5 cells in a dose-dependent manner (Fig 3B and 3C, Table 2), with no effect on either drug observed in parental MES-SA cells.

### R406 modulates MDR by inhibiting P-gp drug transport

**Fig 4:**
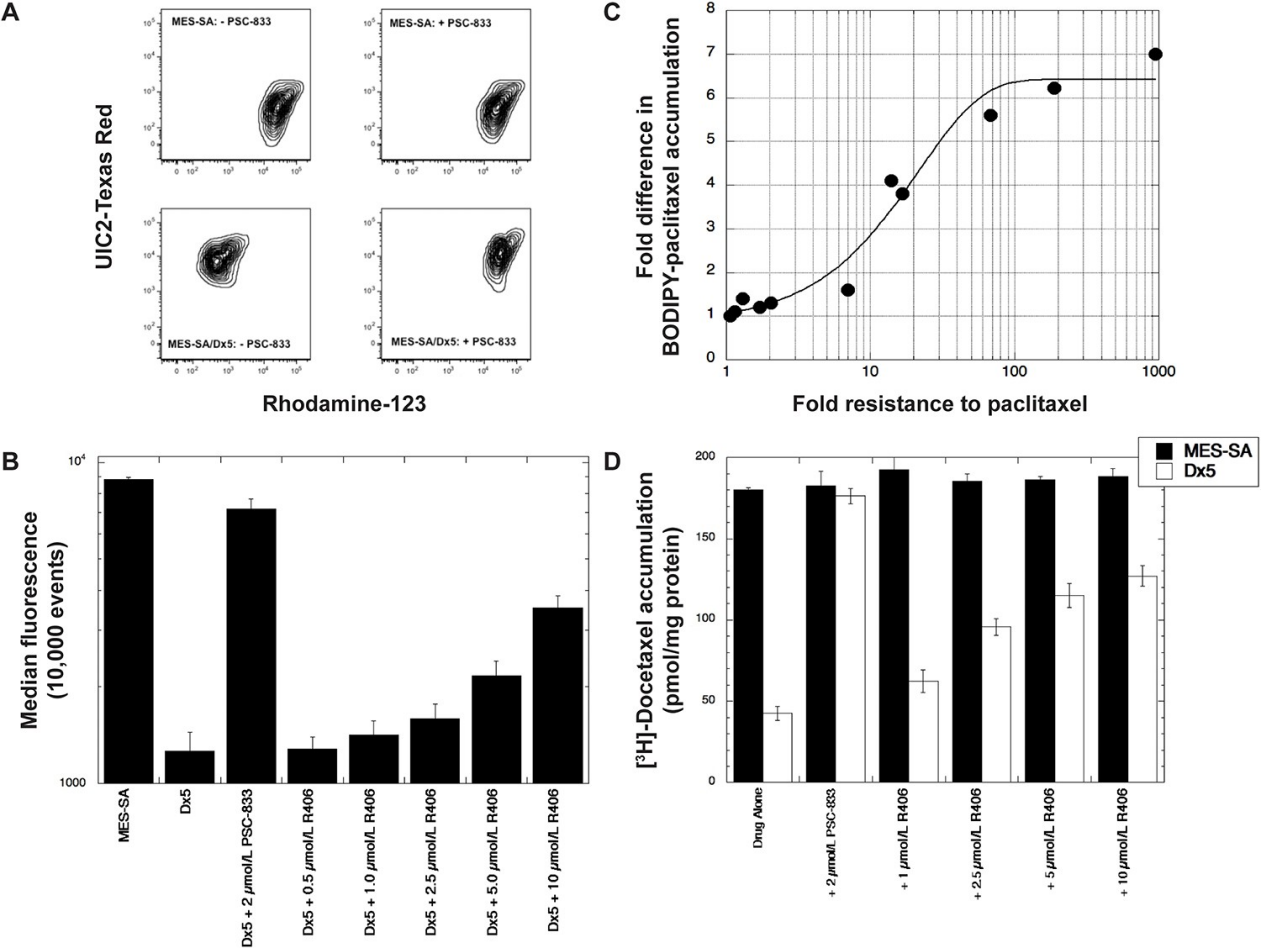
R406 inhibits drug transport in P-gp(+) cells. (A) UIC2-Texas Red staining was used to screen for P-gp expression in MES-SA and MES-SA/Dx5 cells following a 1 h Rhodamine-123 accumulation at 37 °C ± PSC-833 (2 μmol/L). Conditions were measured by flow cytometry. (B) BODIPY-labeled paclitaxel accumulation was determined following a 1 h accumulation ± PSC-833 (2 μmol/L) and R406 (0.5–10 μmol/L). (C) The dose-dependent modulation of paclitaxel resistance (x-axis) by R406 (0.25 to 2.5 μmol/L) is strongly associated with its effect on P-gp inhibition in BODIPY-paclitaxel accumulation assays (y-axis) in the *ABCB1* variants MES-SA/Dx5 and SK-OV-3/TR. P-gp inhibition by 2 μmol/L PSC-833 is included for reference. (D) [^3^H]-docetaxel levels in MES-SA/Dx5 cells were determined following a 1 h accumulation at 37 °C and normalized to protein content. The average of three determinations is presented ± standard deviation.

The functional status of P-gp was assessed by a flow cytometry-based drug accumulation assay using Rhodamine-123, and P-gp expression was determined by UIC2-Texas Red staining (Fig 4A). Following a 1 h drug incubation at 37 °C, parental MES-SA cells had high levels of intracellular Rhodamine-123 (x-axis) and were negative for P-gp (y-axis). Treatment with PSC-833 (2 μmol/L) did not have an effect on Rhodamine-123 levels in parental cells. The MDR MES-SA/Dx5 variant was positive for P-gp and had low levels of intracellular Rhodamine-123, which were restored to parental MES-SA following treatment with PSC-833. We confirmed this finding using BODIPY-labeled paclitaxel under the same experimental conditions and found a significant difference in intracellular drug between MES-SA and its MDR variant, and these levels could be restored to parental levels in the presence of PSC-833 (Fig 4B).

The ability of R406 to inhibit P-gp was tested using several concentrations of the Syk inhibitor up to 10 μmol/L, a concentration that was possible in these short-term assays. As with the other MDR modulators tested, both cell lines were exposed to R406 for 30 min at 37 °C prior to introducing BODIPY-paclitaxel. Flow cytometry revealed a dose-dependent increase in paclitaxel levels in MES-SA/Dx5 cells with 40% MES-SA levels achieved with 10 μmol/L R406 (Fig 4B).

There was a strong association detected between the modulating effects of paclitaxel resistance following R406 treatment at various concentrations in SRB assays and R406’s effect on BODIPY-paclitaxel accumulation in both the MES-SA/Dx5 and SK-OV-3/TR *ABCB1*(+) variants (Fig 4C), indicating that R406 inhibition of P-gp transport was responsible for the sensitization to paclitaxel. Accumulation assays were reproduced using [^3^H]-docetaxel and comparable levels of P-gp inhibition were observed (Fig 4D).

### Treatment with R406 does not affect P-gp expression

**Fig 5:**
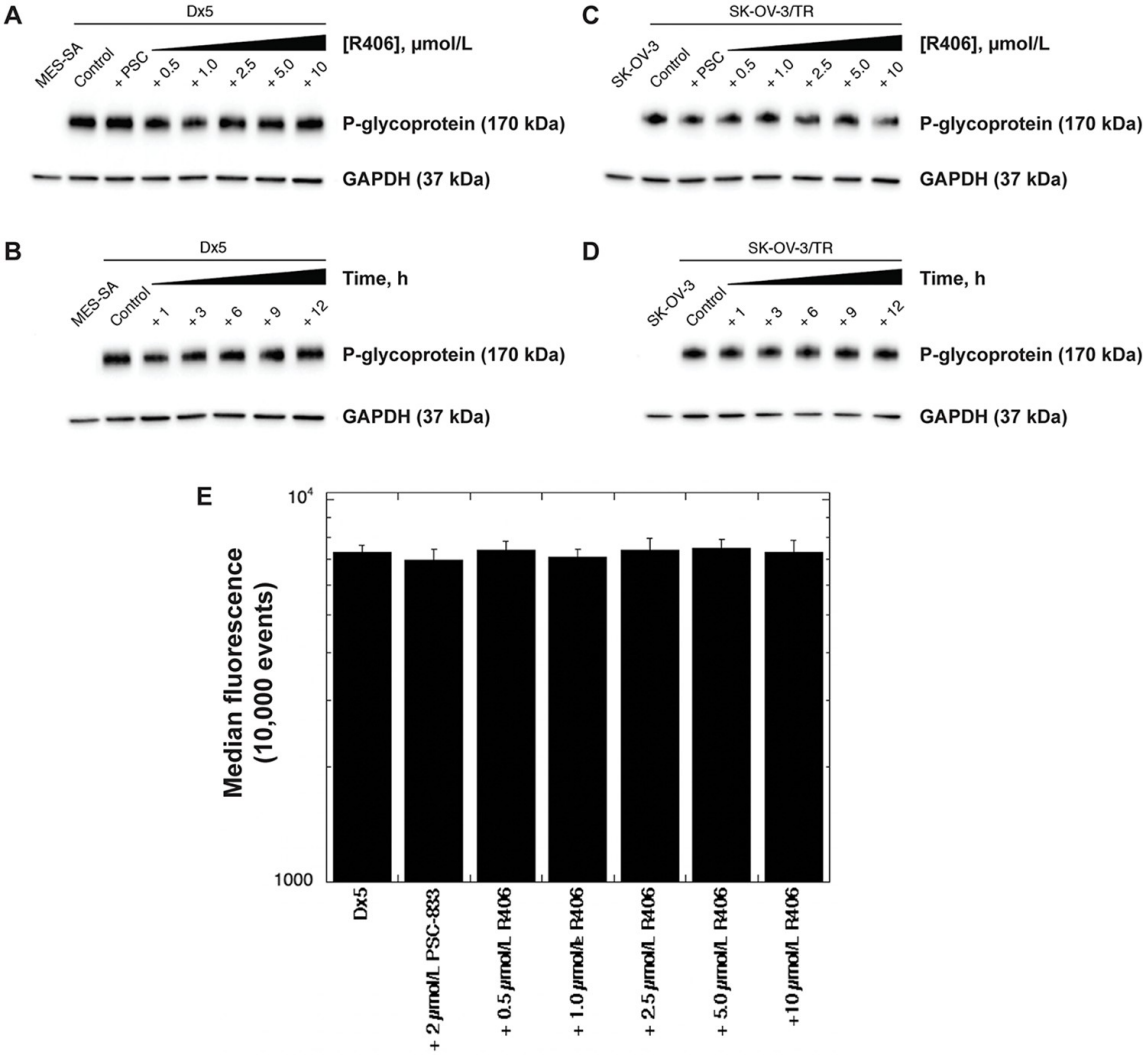
Treatment with R406 does not affect P-gp expression. P-gp expression in MES-SA/Dx5 cells (A) or SK-OV-3/TR (C) cells was determined following a 3 h exposure to R406 (0.5 to 10 μmol/L) compared to an untreated control. MES-SA cells were included as negative control, and cells were exposed to PSC-833 (2 μmol/L) under identical conditions. Cells were harvested and P-gp content determined in total cell lysates as described in the Methods section. P-gp expression was also determined in MES-SA/Dx5 (B) or SK-OV-3/TR (D) cells following exposure to 10 μmol/L R406 after 1, 3, 6, 9, and 12 h. (E) P-gp expression was determined in MES-SA/Dx5 cells in Fig 4B using the UIC2 monoclonal antibody recognized with a Texas Red goat anti-mouse secondary antibody. Data are expressed as the median fluorescence of 10,000 events per condition ± standard deviations.

P-gp expression was determined after treatment with R406 in both MES-SA/Dx5 and SK-OV-3/TR variants. MES-SA/Dx5 cells were exposed to R406 (0.5 to 10 μmol/L) for 3 h, harvested and lysed for immunoblotting with the P-gp-specific monoclonal antibody C219. No difference in P-gp content was observed compared to an untreated control, and cells were exposed to 2 μmol/L PSC-833 as an additional control (Fig 5A). In addition, MES-SA/Dx5 cells were exposed to 10 μmol/L R406 over a time course (1, 3, 6, 9, 12 h) and equivalent P-gp expression was observed relative to the untreated control (Fig 5B). These data were reproduced in the SK-OV-3/TR variant under identical experimental conditions (Fig 5C and 5D). Finally, MES-SA/Dx5 cells in Fig 4B were stained with UIC2-Texas Red to determine P-gp content, and we found that P-gp levels were consistent throughout the experiment (Fig 5E), confirming that R406 inhibits transporter function and not P-gp expression.

### R406 stimulates ATPase activity in P-gp(+) membranes

**Fig 6:**
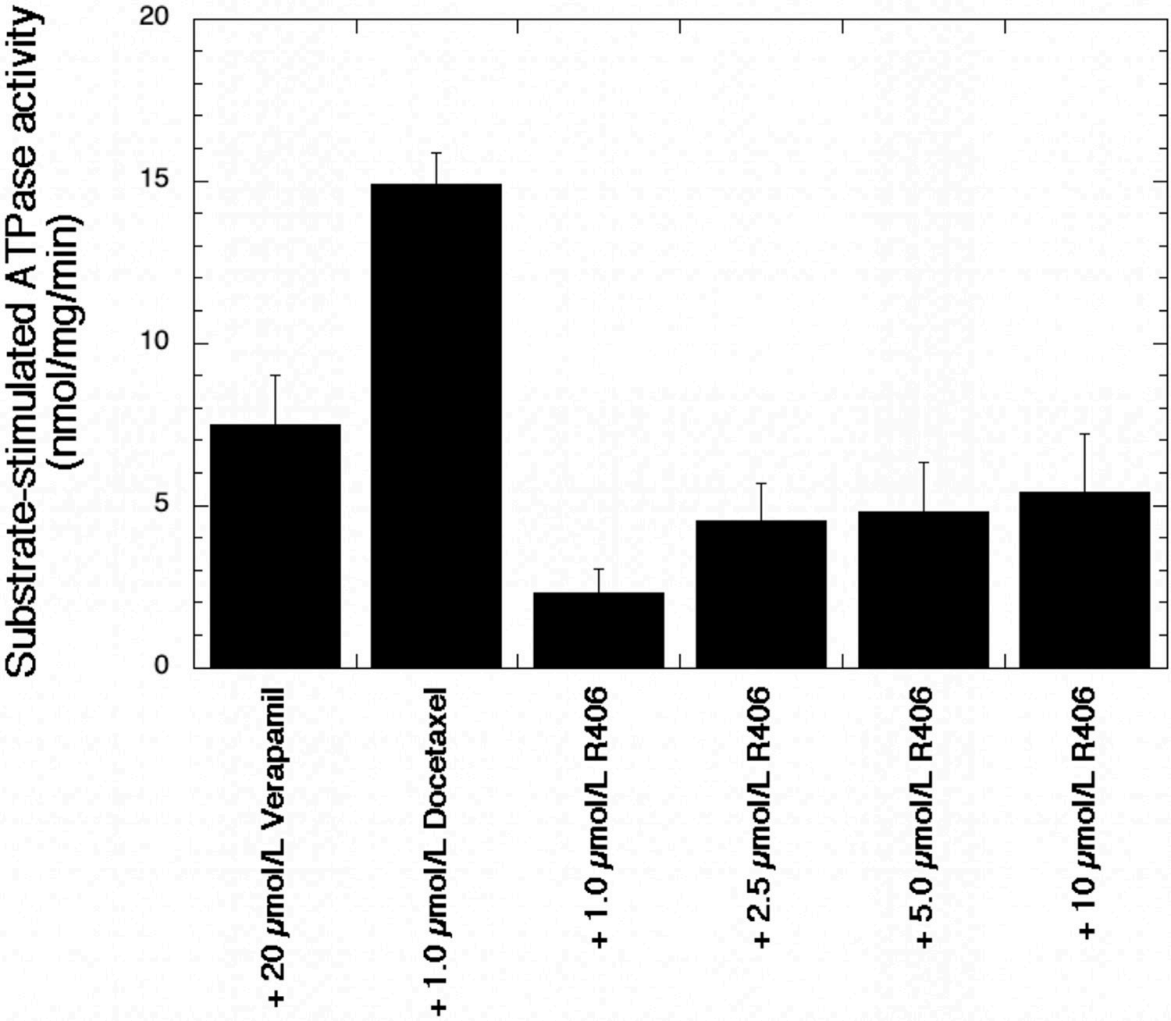
R406 stimulates ATPase activity in P-gp-enriched membranes. The Corning Gentest ATPase assay was used to determine P-gp activity following treatment with R406. Membranes isolated from insect cells expressing P-gp were pre-incubated with R406 (1.0 to 10 μmol/L), verapamil (20 μmol/L) or docetaxel (1 μmol/L) for 5 min at 37 °C, followed by the addition of ATP and an additional 30 min incubation at 37 °C. A colorimetric reagent supplied by the manufacturer was added to each experimental condition and the plate was read at 800 nm. Conditions were run with and without sodium orthovanadate. All data are expressed as the average of triplicate measurements ± standard deviation.

As an indirect assay for P-gp activity, we measured ATPase stimulation after treatment with R406 over a dose range (1.0 to 10 μmol/L) using membranes isolated from insect cells expressing human *ABCB1* cDNA. Vanadate-sensitive ATPase activity was observed with each R406 concentration tested with a maximum of 5.4 nmol/mg/min ATPase activity measured after exposure to 10 μmol/L R406 (Fig 6). Both verapamil (20 μmol/L) and docetaxel (1.0 μmol/L) were included as positive controls for ATPase activity.

## Discussion

Activation of the *ABCB1* gene is a predominant mechanism of resistance in response to selection with taxanes *in vitro* [1–3, 16–19]. In cell models co-selected with P-gp inhibitors, non-MDR mechanisms of taxane resistance emerge including elevated class III beta-tubulin (*TUBB3*) expression [3, 20–22], altered expression of cell cycle regulators such as CDKN1A/p21 and BRCA1 [3, 23–26] and apoptotic regulators [27, 28], and epithelial to mesenchymal transition [3, 29–31].

Elevated Syk expression has also been associated with recurrent high-grade serous ovarian carcinomas compared to primary tumors [9], but its role in malignancies remains controversial. Yu et al. observed elevated Syk in two paclitaxel-selected variants, SK-OV-3/TR and MPSC1/TR, making it an interesting target for functional studies considering the number of small molecule inhibitors currently available. Exposure to the Syk inhibitor R406 resulted in substantial sensitization in both MDR variants, while results with the inhibitors P505-15 and GS-9973 were less impressive as were results in parental cell lines. The stabilization of microtubules as demonstrated by the increase in both acetylated- and detyrosinated (glu)-alpha-tubulin following combination paclitaxel and R406 treatment over paclitaxel alone could be explained by the increase in intracellular drug in the SK-OV-3/TR and MPSC1/TR variants. We were not able to obtain the variant SK-OV-3c.2 used in tumor xenograft experiments with R406 (9), but we recommend testing this cell line for *ABCB1* expression.

We studied the ability of R406 to sensitize another P-gp variant, the doxorubicin-selected MES-SA/Dx5, to taxanes in order to test whether these effects were Syk-independent since both parental MES-SA and its MDR variant are negative for Syk. We confirmed that R406 can modulate not only paclitaxel resistance in the MES-SA/Dx5 variant, but also resistance to other P-gp substrates. Furthermore, this sensitization resulted from the inhibition of transporter function as demonstrated by our accumulation assays. These results were confirmed in assays performed with the *ABCB1*/P-gp(+) SK-OV-3/TR variant, where 2.5 μmol/L R406 completely modulated paclitaxel resistance to parental SK-OV-3 levels. We also found that R406 stimulated vanadate-sensitive ATPase in P-gp(+) membranes. Compared to other known P-gp inhibitors, R406 is a modest P-gp modulator in MES-SA/Dx5, with 42% inhibition at 10 μmol/L R406 in MES-SA/Dx5, the highest concentration achieved in our drug accumulation assays.

Our experiments targeting Syk function indicate that inhibition resulting from either RNAi or R406 does not affect taxane activity. We present data in the OVCAR-3 cell line that expresses the most total and p-Syk in our panel of human ovarian cancer models, and taxane activity was not influenced by Syk inhibition. We also have developed non-MDR variants derived from the two Syk(+) cell lines selected with taxanes (paclitaxel or docetaxel) in the presence of PSC-833 to prevent the activation of the *ABCB1* gene. The resistance phenotype in these variants is associated with a reduction in Syk content.

## Conclusion

Our data indicate that neither R406 nor anti-*SYK* siRNA have any direct antitumor activity against the Syk-expressing ovarian cancer lines. Any effect resulting from R406 treatment added to paclitaxel would result from P-gp modulation and not Syk function.
